# Swine Influenza Viruses Isolated from 2019 to 2022 in Shandong Province, China, Exemplify the Dominant Genotype

**DOI:** 10.3390/genes15070849

**Published:** 2024-06-27

**Authors:** Yuzhong Zhao, Lebin Han, Haotian Sang, Sidang Liu, Pingping Yang, Yanmeng Hou, Yihong Xiao

**Affiliations:** College of Animal Science and Veterinary Medicine, Shandong Agricultural University, 61 Daizong Street, Tai’an 271018, China; zyz3578@163.com (Y.Z.); hanlebin867@163.com (L.H.); 18853851797@163.com (H.S.); liusidang@126.com (S.L.); ppyang@sdau.edu.cn (P.Y.); lemonhym@163.com (Y.H.)

**Keywords:** swine influenza viruses, G4 genotype, H1N1, pathogenicity, mice

## Abstract

Swine influenza viruses (SIVs) have been circulating in swine globally and are potential threats to human health. During the surveillance of SIVs in Shandong Province, China, from 2019 to 2022, 21 reassortant G4 genotype Eurasian avian-like (EA) H1N1 subtypes containing genes from the EA H1N1 (HA and NA), 2009 pandemic (pdm/09) H1N1 virus (PB2, PB1, PA, NP, and M), and classical swine (CS) H1N1 (NS) lineages were isolated. The analysis of the key functional amino acid sites in the isolated viruses showed that two mutation sites (190D and 225E) that preferentially bind to the human α2-6 sialic acid receptor were found in HA. In PB2, three mutation sites (271A, 590S, and 591R) that may increase mammalian fitness and a mutation site (431M) that increases pathogenicity in mice were found. A typical human signature marker that may promote infection in humans, 357K, was found in NP. The viruses could replicate efficiently in mouse lungs and turbinates, and one of the H1N1 isolates could replicate in mouse kidneys and brains without prior adaption, which indicates that the viruses potentially pose a threat to human health. Histopathological results showed that the isolated viruses caused typical bronchopneumonia and encephalitis in mice. The results indicate that G4 genotype H1N1 has potential transmissibility to humans, and surveillance should be enhanced, which could provide important information for assessing the pandemic potential of the viruses.

## 1. Introduction

Swine influenza is a highly contagious respiratory disease in pigs caused by influenza A virus (IAV). IAV can infect numerous animal hosts, including humans, birds, dogs, cats, pigs, horses, ferrets, minks, and seals [1]. The viral reservoir and gene pool are maintained in metapopulations of aquatic wild birds [2]. In general, IAV has high host specificity. However, genome segments can overcome the barrier through point mutations (antigenic drift) and gene reassortment (antigenic shift). The past three influenza outbreaks, including Asian influenza in 1957, Hong Kong influenza in 1968, and swine-origin H1N1 influenza in 2009, have been associated with gene reassortants derived from human and animal influenza viruses [3,4,5]. Although many IAV subtypes have been found in pigs, including H1N1, H1N2, H3N1, H3N2, H5N1, H6N6, H9N2, H10N8, and other subtypes of IAV, the H1N1, H1N2, and H3N2 subtypes are consistently isolated from swine herds globally [6,7,8,9,10,11,12,13,14]. Currently, there are four lineages circulating in China swine: the classical swine (CS) H1N1 lineage, the Eurasian avian-like (EA) H1N1 lineage, the 2009 pandemic (pdm/09) H1N1 lineage, and the North American triple-reassortant (TR) lineage [15]. Swine are considered “mixing vessels” for human and animal influenza viruses; therefore, strains with pandemic potential may be produced during mixed infections [16,17]. Improving the timely monitoring of SIVs would facilitate the assessment of the potential risks of such novel reassortant viruses to humans, in addition to their prediction and forewarning.

SIVs typically do not infect humans; however, occasionally, they can cause human infections [18,19,20,21]. The first reported case of a human infected with swine influenza was in 1958 [22]. Although the spread of SIVs between pigs and humans has been reported, so far, only pdm/09 H1N1 has been shown to be able to efficiently transmit and spread across both species. In addition, pdm/09 H1N1 has reassorted with other SIVs, generating viruses with the ability to infect humans, such as a triple-reassortant SIV, which was isolated in Fujian, China, in 2016 [23,24]. This triple-reassortant SIV has also been found in dogs in Guangxi and minks in Shandong [25,26]. In a recently published SIV surveillance study, this triple-reassortant SIV (designated as genotype 4 [G4]) was reported to be the most prevalent in pigs since 2016, and it has all the hallmarks of being highly adapted to infect humans [27].

In the present study, G4 genotypes of SIV were isolated during the epidemiological surveillance of swine herds in Shandong, China, from 2019 to 2022. Genetic characterization and pathogenicity were studied. The isolated G4 genotype strains harbored amino acid sites associated with potential transmissibility to humans, which emphasizes the importance of the timely surveillance of an emerging virus to understand its epidemiological characteristics and genetic evolution to inform the development of swine influenza prevention and control measures.

## 2. Materials and Methods

### 2.1. Ethics Statement

This study was approved by the Ethics Committee of Shandong Agricultural University (Tai’an, China). The project identification code is 2017-041, which was approved on 12 April 2017 (12/04/2017). All animal experiments were carried out in accordance with the guidelines of the Animal Care and Use Committee of Shandong Agricultural University.

### 2.2. Sample Collection and Treatment

A total of 7936 samples (nasal swabs = 7458; lung = 298; and trachea = 180) were collected from different intensive pig farms located in Linyi, Rizhao, Taian, Weifang, Liaocheng, Heze, Yantai, Dezhou, and Jinan in Shandong Province, China, between January 2019 and December 2022 (Table 1). The samples were collected in phosphate-buffered saline (PBS) with 2000 units/mL penicillin and 2000 units/mL streptomycin. The samples were homogenized and centrifuged at 5000× *g* for 5 min at 4 °C. The supernatant was then inoculated into 9-to-10-day-old specific pathogen-free (SPF) embryonated chicken eggs via the allantoic cavity (0.2 mL/embryo). After two passages in embryonated chicken eggs, the allantoic fluids were harvested and tested using a hemagglutination (HA) test with 1% chicken red blood cells, and the H1, H3, H5, and H9 subtypes of influenza viruses were confirmed by RT-PCR. Samples positive for SIV were stored at −70 °C until further use.

### 2.3. Nucleotide Sequencing

Viral RNA was extracted from 200 µL of virus-infected allantoic fluid using TRIzol reagent (Invitrogen, Carlsbad, CA, USA) according to the manufacturer’s instructions. The RNA was reverse-transcribed into cDNA by Uni12 universal primers (5′-AGCAAAAGCAGG-3′) using the ReverTra Ace qPCR RT Kit (TOYOBO, Osaka, Japan). PCR was performed using 2× Rapid Taq Master Mix (Vazyme Biotech Co., Ltd., Nanjing, China). The complete viral genome was amplified using a total of 15 pairs of primers. All PCR products were gel-purified using the QIAquick gel extraction kit (Qiagen, Hilden, Germany). DNA sequencing and primer synthesis were carried out by Sangon Biotech (Shanghai) Co., Ltd., Shanghai, China.

### 2.4. Analysis of Viral Sequencing

The nucleotide sequences were edited using the SeqMan module of the DNAStar package (version 8; DNASTAR, Madison, WI, USA). The genetic similarity calculation and amino acid comparisons were performed using MegAlign in the DNAstar package (version 8; DNASTAR, Madison, WI, USA). The phylogenetic trees were constructed using MEGA 7.0 based on the neighbor-joining model with 1000 replicates. Potential glycosylation sites were identified using the NetGlyc 1.0 Server and consisted of NXS/T sites, where X denotes any amino acid, excluding proline (https://services.healthtech.dtu.dk/, accessed on 16 March 2024).

### 2.5. Virus Titer

Virus titration was determined using 9-to-10-day-old SPF embryonated chicken eggs and is presented as 50% egg infectious titers (EID_50_). Briefly, a series of 10-fold dilutions of infected allantoic fluid was prepared in sterile PBS, and each dilution (0.1 mL) was inoculated into five 9-to-10-day-old SPF embryonated chicken eggs. After 48 h of incubation at 37 °C, the allantoic fluid was harvested from each egg and tested for the presence of HA activity using the HA test. The EID_50_ values were calculated using the Reed–Muench method.

### 2.6. Infection Studies in Mice

To evaluate the pathogenicity of isolates in mice, eight-week-old SPF female BALB/c mice were divided randomly into four groups (eight mice per group). After being anesthetized lightly with dry ice, the mice were intranasally inoculated with the corresponding viruses at a dose of 10^6^ EID_50_ in a 50 µL volume. Additionally, eight mice inoculated with 50 μL of PBS served as negative controls. Three mice in each group were euthanized at 3 days post-infection (dpi), and the lung, turbinate, spleen, kidney, and brain were collected for viral titration. The viral titer was determined in 9-to-10-day-old SPF embryonated chicken eggs, and the EID_50_ values were calculated using the Reed–Muench method. Parts of the tissue samples were fixed in 4% paraformaldehyde, embedded in paraffin, sectioned, and stained with hematoxylin and eosin. Survival and body weight were monitored for 14 days, and mice were euthanized humanely if more than 25% of the initial body weight was lost.

## 3. Results

### 3.1. Virus Isolation and Identification

A total of 7936 clinical samples collected from nine pig farms in Shandong from 2019 to 2022 were detected. Of these, 38 samples were hemagglutination-positive (0.47%), from which 21 viruses were successfully recovered. Among the 21 isolates, 15 were isolated from nasal swab samples, 4 from lung samples, and 2 from trachea samples. The isolation rates from swine in 2019, 2020, 2021, and 2022 were 38.10% (8/21), 28.57% (6/21), 19.05% (4/21), and 14.29% (3/21), respectively (Table 1). The whole genomes of all 21 isolates were sequenced and analyzed.

### 3.2. Phylogenetic Analysis

**Figure 1:**
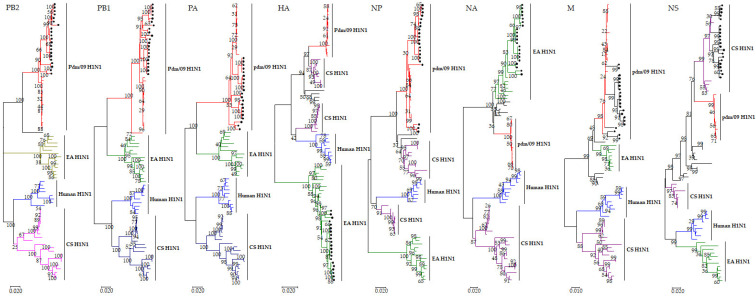
Phylogenetic trees of the PB2, PB1, PA, HA, NP, NA, M, and NS genes of H1N1 subtypes. The trees were generated with MEGA 7.0 using neighbor-joining analysis, and the reliabilities of the trees were assessed by bootstrap analysis with 1000 replicates. The isolates in this study are indicated by black circles.

To investigate the genetic characteristics of the 21 isolated H1N1 viruses, all eight gene segments of the viruses were sequenced and analyzed phylogenetically. Phylogenetic analysis revealed that the hemagglutinin (HA) gene and neuraminidase (NA) gene of the 21 isolated H1N1 viruses belong to the EA H1N1 lineage (Figure 1). The HA genes of the viruses shared a 93.6–100% identity at the nucleotide level, and the NA genes shared a 93.3–100% identity. The identity of the six internal genes of the polymerase basic protein 2 (PB2), polymerase basic protein 1 (PB1), polymerase acid protein (PA), nucleoprotein (NP), matrix protein (M), and nonstructural protein (NS) genes of the 21 isolated H1N1 viruses were 94.9–100%, 95.7–100%, 95.1–100%, 94.8–100%, 95.0–100%, and 94.5–100%, respectively, at the nucleotide level. The PB2, PB1, PA, NP, and M genes of the 21 isolated H1N1 viruses exhibited the same clustering pattern and were clustered into the pdm/09 H1N1 lineage (Figure 1). The NS genes of the 21 isolated H1N1 viruses are clustered together, and they belong to the CS H1N1 lineage (Figure 1). Based on phylogenetic analyses, 21 isolated H1N1 viruses had the same genetically assembled form as the potentially pandemic G4 genotype virus.

### 3.3. EA H1N1 SIV Genotypes

To determine the genotypes of isolated SIVs, a total of 86 sequences of H1N1 subtype SIVs from 2008 to 2021 in Shandong Province were downloaded from the National Center for Biotechnology Information (NCBI) Influenza Virus Resource (http://www.ncbi.nlm.nih.gov/genomes/FLU/FLU.html accessed on 16 March 2024) and the Global Initiative for Sharing All Influenza Data (GISAID) database (http://www.gisaid.org), accessed on 24 December 2023. Duplicate sequences in both databases were removed by matching strain names using Bioedit v7.1.3.0 (Ibis Biosciences, Carlsbad, CA, USA). A phylogenetic analysis of the 21 isolates and 44 EA H1N1 reference strains based on the whole genome was performed (Table 2). The sequences were categorized into three genotypes (A, B, and C). All eight genomic segments of the EA H1N1 subtype isolated in Shandong from 2008 to 2013 were the avian-derived subtype, designated as genotype A. From 2014 to 2019, two distinct genotypes were characterized as triple-reassortant. Notably, EA H1N1 in genotype B was a novel triple-reassortant containing the HA, NA, and M genes of EA H1N1, the NS gene from CS H1N1, and the four remaining gene segments from pdm/09 H1N1. From 2014 to 2015, all isolated viruses belonged to genotype B. In 2016, a novel triple-reassortant SIV of genotype C was isolated. The reassortant contained the HA and NA genes derived from EA H1N1, the NS gene from CS H1N1, and the five remaining gene segments from pdm/09 H1N1. After 2016, genotype C dominated the pig population. Genotyping of the 65 SIV isolates revealed that genotype C was the most common (64.61%), followed by genotype B (26.15%) and genotype A (9.23%). All viruses isolated in Shandong Province from 2019 to 2022 belonged to genotype C.

### 3.4. Molecular Characterization

The molecular characteristics of all 21 isolated H1N1 viruses were analyzed based on the whole-genome sequences. Based on the deduced amino acid sequences of the HA genes, all 21 isolated H1N1 viruses possessed a single basic amino acid (PSIQSR↓G or PSIQSK↓G) in the HA cleavage site, which is characteristic of low-pathogenic avian influenza viruses (Table 3). Five potential glycosylation sites on HA (positions 14 (NST), 26 (NVT), and 277 (NCT) in HA1 and positions 484 (NGT) and 583 (NGS) in HA2) were predicted in all the isolates. For HA genes, 190D was found in 10 isolates, and 225E was found in 14 isolates, indicating that it may bind efficiently to the human α2-6 sialic acid-linked receptor [28,29]. The 225E of HA enhances the transmission of EA H1N1 SIV in guinea pigs [30]. 225E was found in the HA of 14 isolates. The 158 sites in the HA gene of all isolates were G, which may affect the antigenicity of the virus [31]. The 425M increased the replication and pathogenicity of SIV in mice [32]. All 21 isolated H1N1 viruses were not mutated at position 425. All 21 isolated H1N1 viruses had 271A and 590/591 SR in the PB2 gene, suggesting their ability to adapt to the mammalian host [33]. The 251K in the PB2 gene of EA H1N1 SIV enhances viral replication and pathogenicity in mice [34]. Among the isolates, only three strains possessed the 251K. All 21 isolates had 431M in the PB2 gene, which enhances the virulence of EA H1N1 SIV in mice [35]. The 627K and 701N affect the host range and virulence of influenza viruses [36,37,38]. None of the isolates had mutations at positions 627 and 701. The 309N enhanced the replication and pathogenicity of SIVs in mice [32]. None of the isolates exhibited mutations at this site. The 100I, 321K, 330V, and 639T enhance the pathogenicity and transmissibility of the EA H1N1 SIV [39]. None of the isolates had mutations at these positions. The amino acid substitutions associated with reduced susceptibility to NA inhibitors were not observed in all the isolated H1N1 viruses, suggesting that the isolated virus was sensitive to the antiviral drugs oseltamivir and zanamivir [40]. However, all isolates carried 31N in the M2 gene, indicating resistance to amantadine analogs [41]. The 48Q, 98K, and 99K in the NP gene enable the virus to evade human MxA restriction [42]. None of the isolates had mutations at these positions. All 21 isolated H1N1 viruses had 357K in NP, which reportedly enhances the virulence of SIVs in mice [43].

### 3.5. Mouse Experiments

**Figure 2:**
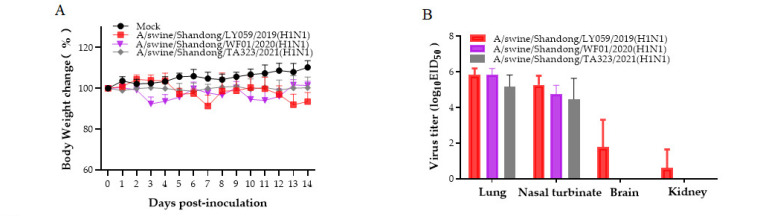

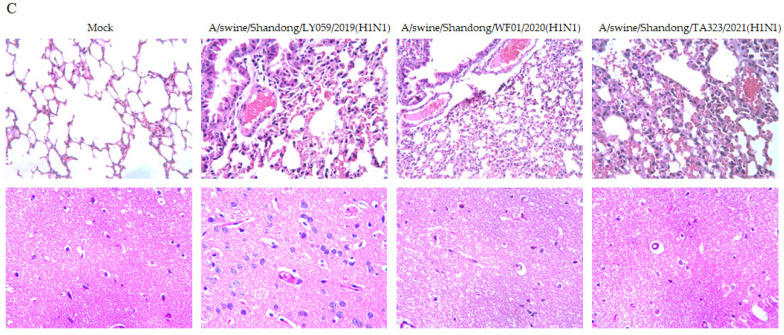
The pathogenicity of the isolates in mice. (**A**). Mouse body weights were monitored daily for 14 days. The values represent the average scores of overall body weight loss compared with the initial body weight ± standard deviation (SD). (**B**). Viral titers in lungs and nasal turbinates of the infected mice (n = 3) after 3 DPI were determined in 9-to-10-day-old SPF embryonated chicken eggs. (**C**). Histopathological analysis of lungs and brains. The lungs or brains of the infected mice were fixed with formalin, embedded in paraffin, stained with hematoxylin and eosin, and observed under a microscope at 200× magnification.

Three isolates (A/swine/Shandong/LY059/2019 (H1N1), A/swine/Shandong/WF01/2020 (H1N1), and A/swine/Shandong/TA323/2021 (H1N1)) were selected to investigate replication and pathogenicity in mice. No significant changes in clinical signs were observed in infected mice. Mice infected with A/swine/Shandong/LY059/2019 (H1N1) had a maximum weight loss of 8.76% at 7 dpi. The virus replicated in the lungs and nasal turbinates of mice with mean titers of 5.83 log10 EID_50_/mL and 5.27 log10 EID_50_/mL, respectively, at 3 dpi. Two out of the three mice revealed detectable virus replication in the brains, and one mouse displayed detectable virus replication in the kidney, with mean titers of 2.65 and 1.8 log10 EID_50_, respectively. Mice infected with A/swine/Shandong/WF01/2020 (H1N1) had a maximum weight loss of 8.76% at 4 dpi. The virus replicated in the lungs and nasal turbinates of mice with mean titers of 5.83 log10 EID_50_/mL and 4.73 log10 EID_50_/mL, respectively, at 3 dpi. There was no significant weight loss in mice infected with A/swine/Shandong/TA323/2021 (H1N1) during the observation period (Figure 2A). The virus replicated in the lungs and nasal turbinates of mice with mean titers of 5.17 log10 EID_50_/mL and 4.47 log10 EID_50_/mL, respectively, at 3 dpi (Figure 2B). Histopathological analysis showed that the lungs of mice showed bronchopneumonia with changes in alveolar wall thickness, inflammatory cell infiltration, capillary congestion, and epithelial cell shedding at 3dpi (Figure 2C). Encephalitis was also observed in infected mice with vascular congestion and meningeal congestion at 3dpi (Figure 2C). The other tissues had no obvious pathological changes at 3 dpi. The results indicate that the isolated virus has low pathogenicity in mice.

## 4. Discussion

The EA H1N1 subtype was first isolated from pigs in Belgium in 1979, after which it spread rapidly in European and Asian countries [44]. In 2001, it was isolated in Hong Kong, China, and has been continuously present in Chinese herds [45]. Since 2005, EA H1N1 SIV has gradually become the dominant spectrum in swine herds in China [15]. After the reintroduction of pdm/09 H1N1 into swine herds, it continued to reassort with the EA H1N1 subtype and gradually replaced the pure EA H1N1 subtype, and it now dominates swine herds in China [44].

The genotypes of isolated viruses in Shandong Province were compiled, and the HA and NA genes of 65 SIVs were observed to belong to the EA H1N1 lineage, excluding 2 CS H1N1 SIVs in 2008 and 6 pdm/09 H1N1 SIVs in 2009 and 2010. In addition, no pure EA H1N1 SIVs were isolated in Shandong after 2013; however, pure EA H1N1 SIVs could still be isolated in some provinces [46]. The observations suggest that the transmission of swine influenza is geographically specific. Notably, fixed genotype B and genotype C appeared after 2014, and genotype C dominated the pig population. In the present study, 21 H1N1 subtypes, all of which belonged to genotype C, were isolated during epidemiologic surveillance in Shandong Province. The form of this genotype is identical to that of the potentially pandemic G4 EA H1N1 subtype. Previous studies have shown that this G4 EA H1N1 subtype exhibits efficient infectivity and transmission in ferret models; in addition, serologic surveillance has revealed that G4 EA H1N1 subtype infection rates can reach 10.4% and 4.4% in pig workers and the general population, respectively, indicating that the G4 EA H1N1 subtype has acquired a higher human infectivity [14]. The isolate belongs to the same genotype virus as the already reported human isolates A/Fujian-cangshan/SWL624/2016 (H1N1), A/Tianjin-baodi/1606/2018(H1N1), and A/Yunnan-Mengzi/1462/2020(H1N1) [23,24,47]. It was reported that the antibodies produced after vaccination with the current World Health Organization (WHO)-recommended vaccine strain A/Hunan/42443/2015(H1N1) did not provide effective protection against A/Yunnan-Mengzi/1462/2020(H1N1), suggesting that a new vaccine strain should be urgently constructed [9]. In addition, the G4 EA H1N1 subtype was isolated from dogs in Guangxi and from mink in Shandong, demonstrating that it has achieved cross-species transmission from pigs to dogs and mink [25,26]. The isolation of this genotype in humans, pigs, dogs, and mink-like species suggests that it has an extremely strong ability to spread across species and that this genotype is exhibiting increasing trends, making it particularly important for epidemiological surveillance and molecular evolutionary analysis.

Our analysis of the key functional amino acid substitutions of the isolated viruses revealed that HA has the 190D (10/21) and 225E (14/21) mutation sites, indicating that it can preferentially bind to the human α2-6 sialic acid receptor and cause human infection [28,29]. In addition, PB2 has the 271A and 590/591 SR (21/21) mutation sites, which may increase mammalian adaptability. PB2 has the 431M (21/21) mutation site and is capable of increasing pathogenicity in mice [35]. The NP has the 357K (21/21) mutation site, a typical human marker that may promote infection in humans [43]. The analysis of resistance sites showed that the isolated viruses were resistant to amantadine [41] and sensitive to NA inhibitors [40]. In conclusion, since mutations generated by SIV during evolution can increase adaptation, pathogenicity, and anti-influenza drug resistance in mammals, these molecular features should be monitored and studied in a timely manner.

Mice are a conventional animal model for studying SIV pathogenesis [48]. All three isolated viruses were able to replicate in the lungs and nasal turbinates of mice without prior adaptation. Among other results, virus titers indicated that the three isolated viruses replicated better in the lungs than in turbinates. The results showed that isolates could replicate better in the lower respiratory tract than in the upper respiratory tract. In addition, A/swine/Shandong/LY059/2019 (H1N1) can replicate not only in the lungs and nasal turbinates of mice but also in the brains and kidneys of mice. In our previous study, this genotypic virus did not replicate in the kidneys and brains of mice; however, our latest isolate appeared to replicate in the kidneys and brains of mice, highlighting the progressive increase in the infectivity of this genotypic strain. The exact replication mechanism in the kidney and brain remains to be further investigated. Histopathological analysis revealed changes in the lungs of mice, with alveolar wall thickening, inflammatory cell infiltration, capillary congestion, and epithelial cell detachment. In addition, vascular congestion and meningeal congestion changes were observed in the brain.

## 5. Conclusions

In view of the potential transmissibility of viruses from pigs to humans, the surveillance of influenza viruses transmitted between swine and human populations should be intensified. In addition, further research on virulence factors influencing influenza viruses is required to further assess their threat to public health.

## Figures and Tables

**Table 1:** Detailed information on samples collected for SIV isolates.

Strain Name	Subtype	Date	Cities	HA Titer	Clinical Signs
A/swine/Shandong/LY059/2019	H1N1	01/2019	Linyi	2^7^	Fever, coughing, sneezing
A/swine/Shandong/TA002/2019	H1N1	02/2019	Taian	2^7^	NO
A/swine/Shandong/TA003/2019	H1N1	02/2019	Taian	2^7^	NO
A/swine/Shandong/TA004/2019	H1N1	02/2019	Taian	2^7^	NO
A/swine/Shandong/TA005/2019	H1N1	02/2019	Taian	2^7^	Fever, coughing
A/swine/Shandong/TA006/2019	H1N1	11/2019	Taian	2^6^	NO
A/swine/Shandong/TA008/2019	H1N1	11/2019	Taian	2^6^	NO
A/swine/Shandong/LW020/2019	H1N1	11/2019	Jinan	2^6^	Coughing, sneezing
A/swine/Shandong/WF01/2020	H1N1	05/2020	Weifang	2^8^	Coughing, sneezing
A/swine/Shandong/DZ01/2020	H1N1	05/2020	Dezhou	2^6^	NO
A/swine/Shandong/TA153/2020	H1N1	11/2020	Taian	2^6^	NO
A/swine/Shandong/HZ1766/2020	H1N1	11/2020	Heze	2^8^	NO
A/swine/Shandong/TA2065/2020	H1N1	12/2020	Taian	2^8^	NO
A/swine/Shandong/TA3812/2020	H1N1	12/2020	Taian	2^6^	NO
A/swine/Shandong/LY4407/2021	H1N1	01/2021	Linyi	2^7^	NO
A/swine/Shandong/TA4500/2021	H1N1	03/2021	Taian	2^7^	Fever, coughing, sneezing
A/swine/Shandong/TA323/2021	H1N1	12/2021	Taian	2^7^	Fever, coughing, sneezing
A/swine/Shandong/TA6274/2021	H1N1	12/2021	Taian	2^7^	NO
A/swine/Shandong/TA6303/2022	H1N1	03/2022	Taian	2^7^	NO
A/swine/Shandong/TA6417/2022	H1N1	04/2022	Taian	2^7^	NO
A/swine/Shandong/TA6902/2022	H1N1	12/2022	Taian	2^6^	NO

NO: No obvious symptoms.

**Table 2:** Genotypes of H1N1 SIV in Shandong Province, China, from 2008 to 2021.

Strain Name	Genotype	Lineage Assigned to Gene Segments							
		PB2	PB1	PA	HA	NP	NA	M	NS
A/swine/Shandong/101/2008	A								
A/swine/Shandong/275/2009									
A/swine/Shandong/327/2009									
A/swine/Shandong/436/2012									
A/swine/Shandong/39/2013									
A/swine/Shandong/862/2013									
A/swine/Shandong/S93/2014	B								
A/swine/Shandong/S113/2014									
A/swine/Shandong/S153/2014									
A/swine/Shandong/S269/2014									
A/swine/Zhucheng/90/2014									
A/swine/Shandong/573/2014									
A/swine/Shandong/414/2014									
A/swine/Shandong/807/2014									
A/swine/Shandong/POS136/2015									
A/swine/Shandong/POS2499/2015									
A/swine/Shandong/POS2500/2015									
A/swine/Shandong/POS2545/2015									
A/swine/Taian/23/2017									
A/swine/Shandong/123/2018									
A/swine/Shandong/75/2018									
A/swine/Shandong/60/2019									
A/swine/Shandong/633/2019									
A/swine/Shandong/1203/2016	C								
A/swine/Shandong/1207/2016									
A/swine/Shandong/16/2016									
A/swine/Shandong/36/2016									
A/swine/Shandong/9/2016									
A/swine/Shandong/JM78/2017									
A/swine/Shandong/LY142/2017									
A/swine/Laiwu/16/2017									
A/swine/Shandong/0334/2017									
A/swine/Shandong/0336/2017									
A/swine/Shandong/0337/2017									
A/swine/Shandong/10/2017									
A/swine/Shandong/13/2017									
A/swine/Shandong/209/2017									
A/swine/Shandong/363/2017									
A/swine/Shandong/451/2017									
A/swine/Shandong/540/2017									
A/swine/Shandong/612/2017									
A/swine/Taian/95/2017									
A/swine/Taian/97/2017									
A/swine/China/Qingdao/2018									
A/swine/Shandong/LY059/2019									
A/swine/Shandong/TA002/2019									
A/swine/Shandong/TA003/2019									
A/swine/Shandong/TA004/2019									
A/swine/Shandong/TA005/2019									
A/swine/Shandong/TA006/2019									
A/swine/Shandong/TA008/2019									
A/swine/Shandong/LW020/2019									
A/swine/Shandong/WF01/2020									
A/swine/Shandong/DZ01/2020									
A/swine/Shandong/TA153/2020									
A/swine/Shandong/TA1766/2020									
A/swine/Shandong/TA2065/2020									
A/swine/Shandong/TA3812/2020									
A/swine/Shandong/LY4407/2021									
A/swine/Shandong/TA4500/2021									
A/swine/Shandong/TA5208/2021									
A/swine/Shandong/TA6274/2021									
A/swine/Shandong/TA6303/2022									
A/swine/Shandong/TA6417/2022									
A/swine/Shandong/TA6902/2022									
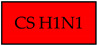 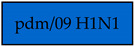 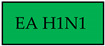									

**Table 3:** Functional amino acid substitutions in the isolated viruses.

Gene Segments	Amino Acid Mutations	21 H1N1 Isolates
**HA** ^a^		
Allows efficient binding to human α2–6 sialic acid receptors	190D, 225D/E [28,29]	190D (10), 190V (9),190N (2); 225G (6), 225E (14), 225K (1)
Increased the transmissibility of EA H1N1 SIV in guinea pigs	D225E [30]	225E (14), 225G (6), 225K (1)
Influences the antigenic properties of EA H1N1 SIV	E158G [31]	158G (21)
Increased the replication and pathogenicity of SIV in mice	L425M [32]	425N (21)
**PB2**		
A combination of 271A with the 590/591 SR polymorphism is critical for SIVs for efficient replication and adaptation in mammals	T271A-A590S-A591R [33]	271A (21)-590S (21)-591R (21)
Increased viral replication and pathogenicity of the EA H1N1 SIV	R251K [34]	251R (18), 251K (3)
Increased the virulence of EA H1N1 SIV in mice and contributed to high polymerase activity and viral genome transcription and replication	T431M [35]	431M (21)
Influences the host range and virulence of influenza viruses	627K, 701N [36,37,38]	627E (21), 701D (21)
Increased the replication and pathogenicity of SIVs in mice	D309N [32]	309D (21)
**PA**		
Increases the pathogenicity and transmission of EA H1N1 SIV	100I, 321K, 330V, 639T [39]	100V (21), 321N (21), 330I (21), 639A (21)
**NA** ^b^		
Acquired resistance to neuraminidase	I222V, H274Y, R292K, N294S [40]	222I (21), 274H (21), 292R (21), 294N (21)
**M2**		
Resistance to amantadine-based drugs	A26F, V27A, A30T/V, S31N, G34E [41]	26L (21), 27V (20)/27I (1), 30A (21), 31N (21), 34G (21)
**NP**		
Escape Human MxA Restriction	48Q, 98K, 99K [42]	48K (21), 98R (21), 99R (21)
Determines the virulence phenotype in mice	Q357K [43]	357K (21)

^a^ H3 numbering, which is used throughout this work; ^b^ N2 numbering, which is used throughout this work.

## Data Availability

The data that support the findings of this study are included within the article.
